# Activation of STING Pathway Contributed to Cisplatin-Induced Cardiac Dysfunction *via* Promoting the Activation of TNF-α-AP-1 Signal Pathway

**DOI:** 10.3389/fphar.2021.711238

**Published:** 2021-08-17

**Authors:** Lintao Wang, Suya Zhang, Jibo Han, Xiaoyan Nie, Yajun Qi, Yingying Han, Xiong Chen, Chaoyong He

**Affiliations:** ^1^ State Key Laboratory of Natural Medicines, Department of Pharmacology, China Pharmaceutical University, Nanjing, China; ^2^ School of Pharmacy, China Pharmaceutical University, Nanjing, China; ^3^ Department of Cardiology, the Second Affiliated Hospital of Jiaxing University, Jiaxing, China; ^4^ Department of Pharmacy, Institute of Cancer and Basic Medicine (IBMC), Chinese Academy of Sciences, The Cancer Hospital of the University of Chinese Academy of Sciences (Zhejiang Cancer Hospital), Hangzhou, China; ^5^ Department of Endocrinology, The First Affiliated Hospital, Wenzhou Medical University, Wenzhou, China

**Keywords:** stimulator of interferon genes, cisplatin, tumor necrosis factor-α, activator protein 1, cardiotoxicity

## Abstract

Cardiovascular complications are a well-documented limitation of conventional cancer chemotherapy. As a notable side effect of cisplatin, cardiotoxicity represents a major obstacle to the treatment of cancer. Recently, it has been reported that cyclic GMP-AMP synthase (cGAS) stimulator of interferon genes (STING) signaling pathway was associated with the occurrence and development of cardiovascular diseases. However, the effect of STING on cardiac damage caused by cisplatin remains unclear. In this study, cisplatin was shown to activate the cGAS-STING signaling pathway, and deficiency of STING attenuated cisplatin-induced cardiotoxicity *in vivo* and *in vitro.* Mechanistically, the STING-TNF-α-AP-1 axis contributed to cisplatin-induced cardiotoxicity by triggering cardiomyocyte apoptosis. In conclusion, our results indicated that STING might be a critical regulator of cisplatin-induced cardiotoxicity and be considered as a potential therapeutic target for preventing the progression of chemotherapy-associated cardiovascular complications.

## Highlights

➢ STING-TNF-α-AP-1 axis contributed to cisplatin-induced cardiotoxicity by triggering cardiomyocyte apoptosis.
➢ Deficiency of STING attenuated cisplatin-challenged cardiac dysfunction.
➢ STING may be a potential therapeutic target for preventing the progression of chemotherapy-associated cardiovascular complications.

## Introduction

Cardiovascular complications associated with conventional cancer chemotherapy are a global problem (El-Awady et al., 2011; McGowan et al., 2017). Cisplatin (cis-Diaminodichloroplatinum, CDDP), one of the most potent chemotherapeutic agents, has been proven effective against various solid tumors, including sarcoma, soft tissue cancer, bone cancer, muscle cancer, and vascular cancer. However, cisplatin-associated toxicity is a well-documented limitation of conventional cancer chemotherapy (de Jongh et al., 2003; Topal et al., 2018; Ma et al., 2020). Cisplatin-induced cardiotoxicity has been reported to mainly include arrhythmia, acute myocardial infarction, supraventricular tachycardia, and atrial fibrillation (Guglin et al., 2009; Ma et al., 2010; El-Awady et al., 2011). Eventually, cardiac toxicity led to congestive heart failure and sudden cardiac death. Therefore, it is urgent to find an effective strategy to prevent cardiovascular complications associated with conventional cancer chemotherapy.

DNA is an important genetic medium, but it can also induce an innate immune response. DNA exposed to the cytoplasm is recognized by cyclic guanosine monophosphate-adenosine monophosphate (cGAMP) synthase (cGAS), triggering signaling cascades that lead to the production of pro-inflammatory factors and interferons (IFNs) (Burdette et al., 2011; Ablasser et al., 2013; Bai and Liu, 2019). As an important intracellular inflammatory signal regulatory protein, stimulator of interferon genes (STING) can recognize not only the second messenger produced by bacteria or viruses, such as cyclic guanosine monophosphate (cGMP) and cyclic adenosine monophosphate (cAMP) but also cyclic dinucleotide produced by the interaction of intracellular DNA and cGAS (Burdette et al., 2011; Ahn et al., 2012; Ablasser et al., 2013; Hu and Shu, 2020); subsequently, it activates TANK binding kinase 1 (TBK1)-interferon regulatory factor 3 (IRF3) signaling pathway and nuclear factor-κB (NF-κB), which regulate the production of IFNs and inflammatory factors (Ishikawa and Barber, 2008; Fang et al., 2017; Ablasser and Chen, 2019; Zhang et al., 2019). Therefore, STING plays a key role in the inflammatory response. Accumulating evidence has found that STING was associated with acute and chronic diseases (Mao et al., 2017; Luo et al., 2018; Chung et al., 2019; Li et al., 2019; Maekawa et al., 2019; Hu et al., 2020; Zhang et al., 2020). Mao et al. found that *Sting* gene knockout could inhibit endothelial inflammation and improve insulin resistance induced by a high-fat diet in mice (Mao et al., 2017). In addition, STING-mediated inflammation induced by mitochondrial stress has been reported to promote myocardial damage (Sliter et al., 2018). Not only that, the activation of STING is caused by cisplatin-induced tubular inflammation and the progression of acute kidney injury (Maekawa et al., 2019). However, the effect of STING on cisplatin-induced cardiotoxicity is unknown.

In the present study, we investigated the effect of STING in cisplatin-induced cardiotoxicity. Our results showed that cisplatin activated the cGAS-STING signaling pathway, and of note, deficiency of STING attenuated cisplatin-induced cardiotoxicity *in vivo* and *in vitro*. Mechanistically, the STING-tumor necrosis factor-α (TNF-α)- activator protein 1 (AP-1) axis contributed to cisplatin-induced cardiotoxicity by triggering cardiomyocyte apoptosis. Altogether, our results indicated that STING might be a critical regulator of cisplatin-induced cardiotoxicity and be considered as a potential therapeutic target for preventing the progression of chemotherapy-associated cardiovascular complications.

## Materials and Methods

### Materials

Cisplatin and AP-1 inhibitor T5224 were purchased from MedChemExpress (Monmouth Junction, NJ, United States). Cisplatin was dissolved in DMF for *in vitro* experiments and saline for *in vivo* experiments. T5224 was dissolved in DMSO for *in vitro* experiments. Antibodies for p-STING (1:1,000, #72971), STING (1:1,000, #13647), *p*-TBK1 (1:1,000, #5483), TBK1 (1:1,000, #3504), BAX (1:1,000, #2772), p-c-Jun (1:1,000, #3270), c-Jun (1:1,000, #9165), p-c-Fos (1:1,000, #5348), c-Fos (1:1,000, #2250), and TNF-α (1:1,000, 11,948) were purchased from Cell Signaling Technology (Danvers, MA, United States). Antibody for BCL2 (1:5,000, BF9103) was purchased from Affinity Biosciences LTD. (Changzhou, Jiangsu, China). Antibody for α-Tubulin (1:1,000, 11,224) was purchased from Proteintech Group (Chicago, Illinois, United States).

### Cell Culture

HL-1 cells were purchased from the Type Culture Collection of the Chinese Academy of Sciences (Shanghai, China) and incubated in DMEM media with 10% fetal bovine serum, 100 U/ml of penicillin, and 100U/ml of streptomycin at 37°C in a humidified 5%CO_2_ incubator.

### Generation of Sting-Knockout HL-1 Cells Using CRISPR-Cas9

Lentiviral vectors that express guide RNAs (gRNAs: 5′-TAC​TTG​CGG​TTG​ATC​TTA​CC-3′) that target STING were produced by co-transfecting HEK293T cells with a cocktail of gRNA/Cas9-expressing lentiCRISPRv2 and pPAX2 plasmids. Lentiviruses were collected at 48 h after transfection, supplemented with 10% FBS, and stored at −80°C. HL-1 cells were transduced with lentiviral vectors for 24 h in combination with 8 μg/ml polybrene. Then, the lentivirus-containing medium was replaced with fresh DMEM with 10%FBS and cultured for another 24 h. Cells were subjected to puromycin (1 μg/ml) selection to isolate *Sting*-knockout (STING KO) HL-1 cells. LentiCRISPRv2 without gRNA was used as an empty vector for mock transduction.

### Cell Counting Kit-8 Assays

HL-1 cells were seeded into 96-well plates at the density of 5,000 cells per well. CCK8 (10 μL) was added to each well for 2 h at 37°C after incubator with different concentrations of cisplatin overnight. Optical density was measured at 450 nm using a microplate spectrophotometer.

### Animal Experiments

Male WT or STING^−/-^ mice (8 weeks old) were used for experiments. WT C57BL/6J mice were obtained from Beijing Vital River Laboratory Animal Technology Co. Ltd. (Beijing, China). STING^−/-^ mice with C57BL/6J background were purchased from Jackson Lab (Stock#025805). All animal care and experimental procedures were performed following the directives outlined in the Guide for the Care and Use of Laboratory Animals (US National Institutes of Health). Animal care and experimental protocols were approved by the China Pharmaceutical University Animal Policy and Welfare Committee (Nanjing, China; approval no. 2020–11–007).

According to previous studies, the dose of cisplatin covered by the clinical dosage range 10–100 mg/m2 was chosen (Zhao et al., 2018). Mice were randomly divided into four groups: saline group (Saline, n = 6), cisplatin group (CDDP, *n* = 6), STING^−/-^ group (STING^−/-^, *n* = 6), STING^−/-^ + cisplatin group (STING^−/-^ + CDDP, n = 6). Cisplatin (6 mg/kg) in 0.9% normal saline was administered by intraperitoneal injection on the first day, eighth day and 15th day as previously reported (Zhao et al., 2018). On the 22nd day, transthoracic echocardiography was conducted on the mice to examine the cardiac function, and then all the animals were sacrificed under sodium pentobarbital anesthesia. The blood samples and heart tissues were collected.

### Cardiac Function

Transthoracic echocardiography (VisualSonics, Toronto, Canada) was performed to measure cardiac function in a non-invasive manner. The left ventricular internal dimension in diastole (LVIDd), left ventricular end-systolic volume (LVESV), left ventricular internal dimension in systole (LVIDs), left ventricular end-diastolic volume (LVEDV), left ventricular posterior wall thickness at end-diastolic (LVPWd), left ventricular posterior wall thickness at end-systolic (LVPWs), interventricular septal thickness at end-diastolic (IVSd), and interventricular septal thickness at end-systolic (IVSs) were assessed from M-mode images. The equations of fractional shortening (FS) = [(LVIDd - LVIDs)/LVIDd] × 100% and ejection fraction (EF) = [(LVEDV-LVESV)/LVEDV]×100% were respectively used to calculate FS and EF.

### Pathological Staining

The cardiac tissues were collected and fixed in 4% paraformaldehyde and embedded in paraffin. The cardiac tissue sections (5 μm) were stained with hematoxylin and eosin for light microscopy examination (Leica, Germany).

### Terminal Deoxynucleotidyl Transferase Mediated dUTP Nick End Labeling Staining

Cardiac tissue sections or HL-1 cells were stained with TUNEL following the manufacturer’s operating procedures (Yeasen, Shanghai, China). Images were taken with the fluorescence microscope (Leica, Germany).

### Determination of Serum Creatine Kinase Isoenzyme

The serum was collected, and the content of serum creatine kinase isoenzyme (CK-MB) was detected by the corresponding kit purchased from Nanjing Jiancheng Co., Ltd (Nanjing, Jiangsu, China).

### RNA Sequencing

Total RNA of cardiac tissues was prepared with trizol reagent (Invitrogen), and RNA-seq was performed by Major Biotechnology Inc. (Shanghai, China). For data analysis, differentially expressed genes were then identified by using *P*-adjust 0.05 as a cutoff. Heap map analysis and KEGG enrichment analysis were performed with online software (http://geneontology.org/).

### Western Blot Analysis

The cardiac tissue (20–40 mg) or HL-1 cells were lysed, the protein concentration was measured, and each protein’s loading amount was balanced. The protein was separated by SDS gel and then transferred to the PVDF membrane. After 90 min, the membranes were preincubated with 5% non-fat milk and incubated with specific primary antibodies overnight. The second antibody conjugated with horseradish peroxidase was incubated for 1 h and visualized using chemiluminescence reagents.

### 2.12 Real-Time Quantitative PCR

Protocol of RT-qPCR was described in a previous paper (Wang et al., 2016). The primer sequences of interleukin 6 (Il6), chemokine (C-X-C motif) ligand 2 (Cxcl2), FBJ osteosarcoma oncogene (Fos), activating transcription factor 4 (Atf4), JunB proto-oncogene (Junb), tumor necrosis factor-alpha-induced protein 3 (Tnfaip3), chemokine (C-X-C motif) ligand 1 (Cxcl1), and β-actin listed in Table 1 were synthesized with Sangon Biotech Co., Ltd (Shanghai, China). The quantitative analysis of genes was normalized to β-actin.

**TABLE 1:** Primers used for real-time qPCR assay.

Gene	Species	Primers(FW)	Primers(RW)
Il6	Mouse	CCA​AGA​GGT​GAG​TGC​TTC​CC	CTG​TTG​TTC​AGA​CTC​TCT​CCC​T
Cxcl-2	Mouse	CCA​ACC​ACC​AGG​CTA​CAG​G	GCG​TCA​CAC​TCA​AGC​TCT​G
Fos	Mouse	CGG​GTT​TCA​ACG​CCG​ACT​A	TTG​GCA​CTA​GAG​ACG​GAC​AGA
Atf4	Mouse	ATG​GCG​CTC​TTC​ACG​AAA​TC	ACT​GGT​CGA​AGG​GGT​CAT​CAA
Junb	Mouse	TCA​CGA​CGA​CTC​TTA​CGC​AG	CCT​TGA​GAC​CCC​GAT​AGG​GA
Tnfaip3	Mouse	ACA​GTG​GAC​CTG​GTA​AGA​AAC​A	CCT​CCG​TGA​CTG​ATG​ACA​AGA​T
Cxcl1	Mouse	CTG​GGA​TTC​ACC​TCA​AGA​ACA​TC	CAG​GGT​CAA​GGC​AAG​CCT​C
β-actin	Mouse	CCG​TGA​AAA​GAT​GAC​CCA​GA	TAC​GAC​CAG​AGG​CAT​ACA​G

### Enzyme-Linked Immunosorbent Assay

The contents of TNF-α and IL-6 in cardiac tissues or TNF-α in HL-1 cells were detected by the corresponding mouse ELISA kits for TNF-α or IL-6, following the manufacturer’s instructions. First, coat the ELISA plate with 100 μL/well of capture antibody in Coating Buffer overnight at 4°C. The cells were washed 3 times with 250 μL/well Wash Buffer and incubated with 200 μL of ELISA Diluent (1×) at room temperature for 1 h. Next, add 100 μL/well of samples and standard to the appropriate wells and incubate at room temperature for 2 h. Aspirate, wash and then incubate with 100 μL/well diluted Detection Antibody to all wells for 1 h. Afterward, aspirate, wash and then incubate with 100 μL/well of diluted Streptavidin-HRP for 30 min. Finally, the cells were washed and incubated with 100 μL/well of 1× TMB Solution for 15 min. Add 100 μL/well of Stop Solution, and read the plate at 450 nm.

### Statistical Analysis

All measurement data were shown as mean ± SEM. Statistical analyses were performed using GraphPad Pro Prism 6.0 (GraphPad, San Diego, CA). Parametric data were used for the statistical analysis. We used one-way ANOVA followed by multiple comparisons test with Bonferroni correction when comparing data from more than two groups and a Student’s *t*-test when comparing data from two groups. It is considered to be significant when *p* < 0.05.

## Results

### Activation of the Cyclic GMP-AMP Synthase-Stimulator of Interferon Genes Pathway Was Detected in Cisplatin-Induced HL-1 Cells

**FIGURE 1:**
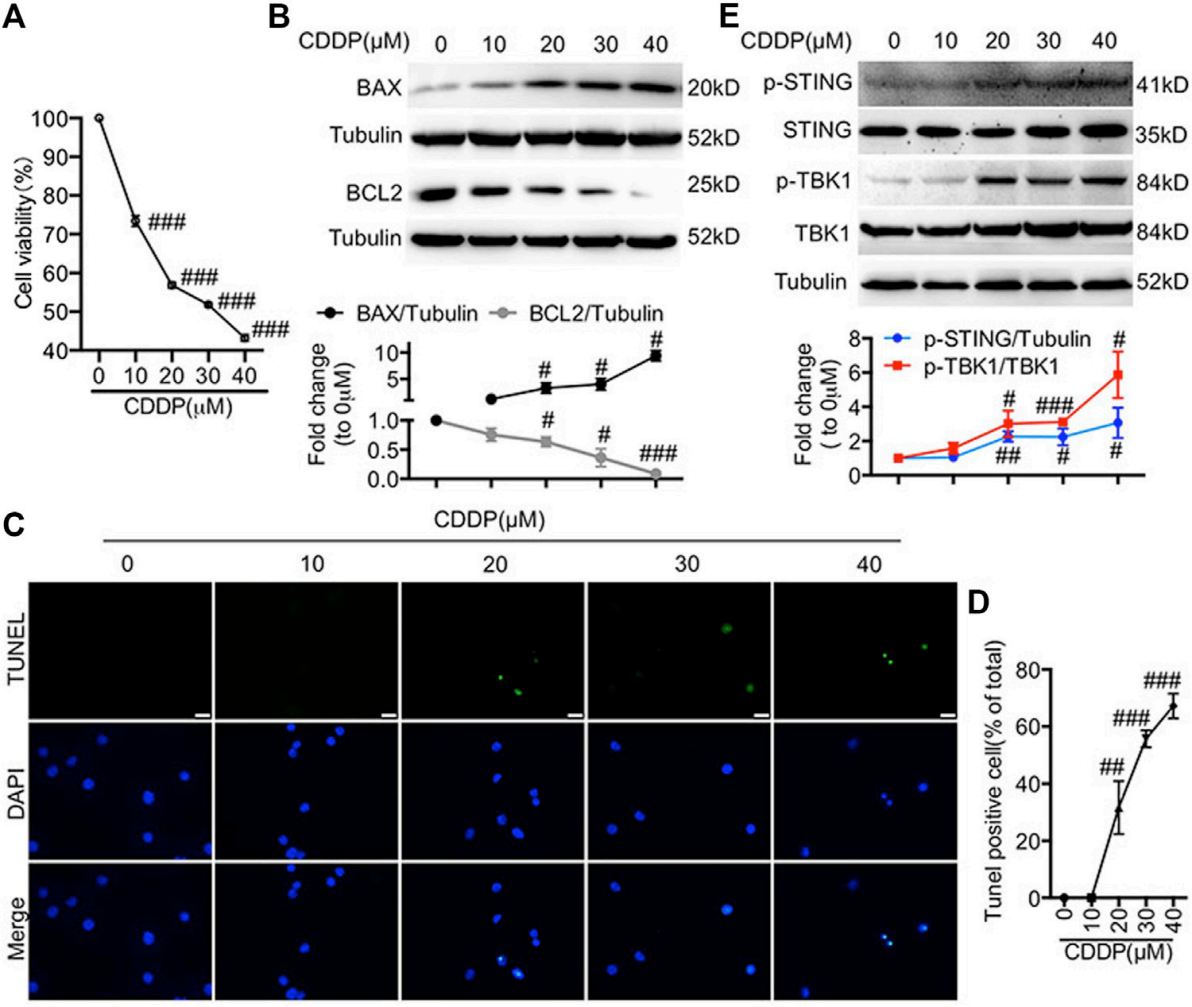
Activation of the cGAS-STING pathway was detected in cisplatin-induced HL-1 cells **(A)** HL-1 cells were incubated with CDDP (10, 20, 30, 40 μM) for 24 h and then the cell viability of HL-1 cells was examined by CCK8 analysis (*n* = 5 independent experiments; ^###^ *p* < 0.001, vs. the 0 group). **(B)** HL-1 cells were incubated with CDDP (10, 20, 30, 40 μM) for 24 h, and then total protein was collected. The protein level of BAX and BCL2 in HL-1 cells was detected by Western blot. (C–D) HL-1 cells were incubated with CDDP (10, 20, 30, 40 μM) for 24 h, and then cell apoptosis induced by CDDP was detected by TUNEL staining. **(C)** Representative images of TUNEL staining in HL-1 cells (Green: TUNEL positive cell, DAPI: nucleus; Scale Bar: 25 μm, ×400 magnification). **(D)** Quantification of TUNEL-positive cells **(E)** HL-1 cells were incubated with CDDP (10, 20, 30, 40 μM) for 1 h, and total protein was collected. The protein level of p-STING, STING, *p*-TBK1, TBK1 in HL-1 cells was detected by Western blot (*n* = 3 independent experiments; ^#^ *p* < 0.05, ^##^ *p* < 0.01, ^###^ *p* < 0.001, vs. Vehicle group).

First, we evaluated the effects of CDDP in HL-1 cells. The IC_50_ (half-inhibitory concentration) value of cisplatin is different in different tumor cell lines, ranging from 10 to 200 μM (Sato et al., 2018; Cesna et al., 2018; Morovati et al., 2019; Kou et al., 2020). In addition, it has been reported that a certain concentration of cisplatin has toxic effects on H9c2 cell line (5 μM) and primary rat cardiomyocytes (200 μM) (Zhang et al., 2017; Qian et al., 2018; Zhao, 2019). Here, the doses of 10, 20, 30, and 40 μM of CDDP were chosen for *in vitro* experiments. Incubation with CDDP (10, 20, 30, 40 μM) for 24 h reduced the viability of HL-1 cells in a dose-dependent manner (Figure 1A). In addition, incubation with CDDP (10, 20, 30, 40 μM) for 24 h resulted in an increased expression of pro-apoptotic BAX and a decreased expression of anti-apoptotic BCL2 (Figure 1B). Furthermore, CDDP induced apoptosis in HL-1 cells (Figures 1C,D). It has been reported that the cGAS-STING pathway participated in myocardial ischemia-reperfusion injury and cardiac hypertrophy (Cao et al., 2018; Hu et al., 2020; Zhang et al., 2020). Notably, the activation of STING and TBK1 was observed in CDDP-challenged HL-1 cells (Figure 1E).

### Suppression of Stimulator of Interferon Genes Ameliorated Cisplatin-Induced Injury in HL-1 Cells

**FIGURE 2:**
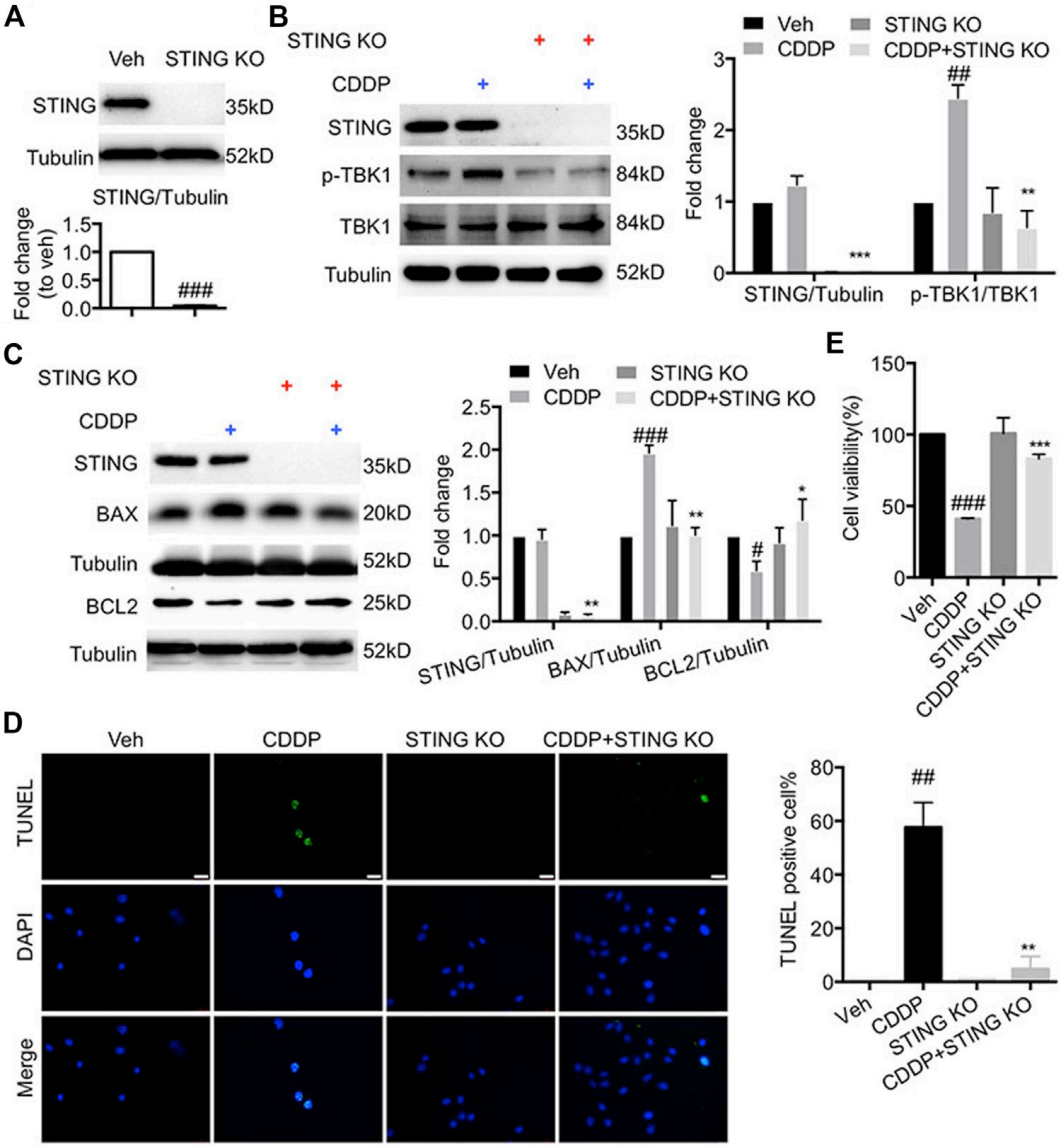
Deficiency of STING ameliorated cisplatin-induced injury in HL-1 cells **(A)** STING was knocked out by CRISP-Cas9. HL-1 cells were subjected to puromycin (1 μg/ml) selection to isolate STING KO cells, and then total protein was collected. The protein level of STING was detected by Western blot. **(B)** HL-1 cells were incubated with CDDP (40 μM) for 1 h, and total protein was collected. The protein levels of *p*-TBK1, TBK1, STING were detected by Western blot. **(C)** HL-1 cells were incubated with CDDP (40 μM) for 24 h, and the protein levels of BAX and BCL2 in HL-1 cells were detected by Western blot. **(D)** Representative images and quantification of TUNEL staining in HL-1 cells. HL-1 cells were incubated with CDDP (40 μM) for 24 h, and cell apoptosis was detected by TUNEL staining (Green: TUNEL positive cell, DAPI: nucleus; Scale Bar: 25 μm, ×400 magnification). **(E)** HL-1 cells were incubated with CDDP (40 μM) for 24 h, and then the cell viability of HL-1 cells was examined by CCK8 analysis (*n* = 3 independent experiments; ^#^ *p* < 0.05, ^##^ *p* < 0.01, ^###^ *p* < 0.001, vs. Vehicle group; ^*^ *p* < 0.05, **p < 0.01, ***p < 0.001, vs. CDDP group).

To confirm the effect of STING on the progression of apoptosis in cisplatin-induced HL-1 cells, we generated the *Sting*-knockout HL-1 cells using CRISPR-Cas9. As shown in Figures 2A,B, CDDP failed to induce the phosphorylation of TBK1 in *Sting*-knockout HL-1 cells. Here, the increased expression of BAX and the reduced expression of BCL2 induced by CDDP were reversed by *Sting* knockout (Figure 2C). Additionally, cardiomyocyte apoptosis and decreased cell viability induced by CDDP were attenuated by *Sting* knockout in HL-1 cells (Figures 2D,E). Altogether, these results indicated that blockage of STING exerted protective effects against CDDP-induced cell injury.

### Deficiency of Stimulator of Interferon Genes Blocked Cisplatin-Induced Cardiac Dysfunction and Injury

**FIGURE 3:**
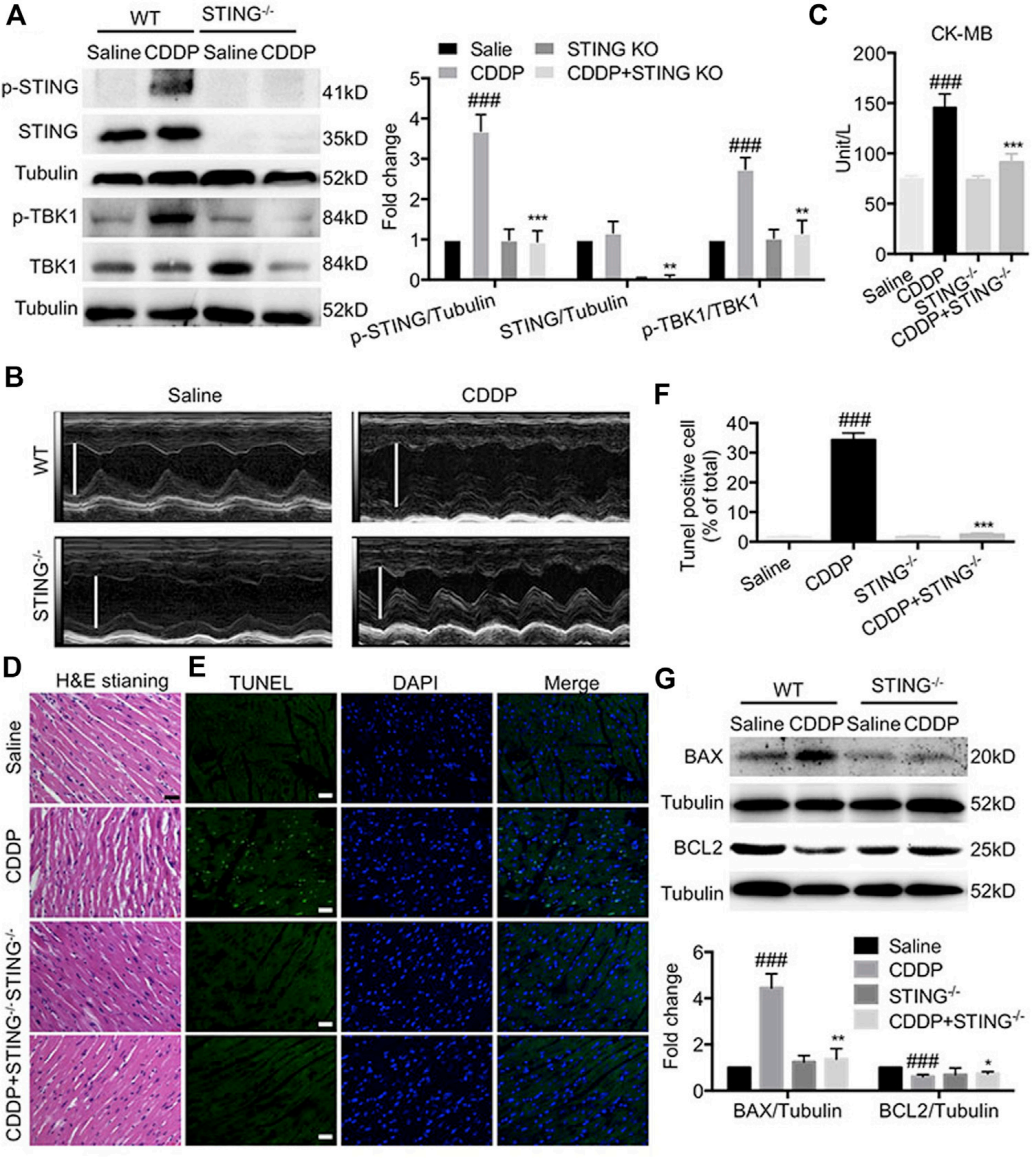
Deficiency of STING blocked cisplatin-induced cardiac dysfunction and injury **(A)** The protein levels of p-STING, STING, *p*-TBK1, TBK1 in cardiac tissues were detected by Western blot. **(B)** Representative images of M-mode of left ventricular **(C)** The levels of serum CK-MB were detected by the corresponding kit. **(D)** Representative images of H and E staining using paraffin section of heart tissues (Scale Bar: 25 μm, ×400 magnification). (E–F) Representative images **(E)** and quantification **(F)** of TUNEL staining using paraffin section of heart tissues. (Green: TUNEL positive cell, DAPI: nucleus; Scale Bar: 25 μm, ×400 magnification) **(G)** The protein levels of BAX and BCL2 in cardiac tissues were detected by Western blot (*n* = 6 in each group; ^###^ *p* < 0.001, vs. Saline group; ^*^ *p* < 0.05, **p < 0.01, ***p < 0.001, vs. CDDP group).

Next, we investigated the role of STING in CDDP-induced cardiotoxicity *in vivo*. Figure 3A showed that the phosphorylation of STING and TBK1 was elevated in cardiac tissues of CDDP-treated mice, but that induced by CDDP was inhibited in STING^−/-^ mice. Furthermore, compared with the saline group, echocardiographic results showed that EF and FS in CDDP-treated mice were notably lowered (Figure 3B and Table 2). Serum CK-MB level was significantly increased by intraperitoneal injection of CDDP. Moreover, the histopathological analysis showed the disruption of cardiac muscle fibers and the loss of striations and intercalated disc induced by CDDP in cardiac tissues. TUNEL staining showed a significant increase in cell death in cardiac tissues of CDDP-challenged mice (Figures 3E,F), and immunoblotting analysis showed an increased expression of BAX and a reduced expression of BCL2 in cardiac tissues of CDDP-treated mice (Figure 3G). However, these changes were reversed by *Sting* knockout in mice (Figure 3; Table 2). These results indicated that deficiency of STING blocked CDDP-induced cardiac dysfunction and injury.

**TABLE 2:** Echocardiographic measurements.

Parameter	Saline	CDDP	STING^−/-^	CDDP + STING^−/-^
N	6	6	6	6
EF (%)	62.36 ± 2.36	47.37 ± 2.00^a^	64.12 ± 2.93	62.90 ± 4.17^b^
FS (%)	33.07 ± 1.64	22.87 ± 1.32^a^	34.47 ± 2.18	32.85 ± 2.80^b^
LVID d(mm)	3.61 ± 0.09	3.86 ± 0.06	3.53 ± 0.09	3.48 ± 0.16
LVID s(mm)	2.41 ± 0.09	2.6 ± 0.23	2.36 ± 0.13	2.38 ± 0.15
LVPW d(mm)	0.76 ± 0.03	0.67 ± 0.04	0.85 ± 0.03	0.73 ± 0.07
LVPW s(mm)	1.25 ± 0.07	0.96 ± 0.06^a^	1.36 ± 0.08	1.16 ± 0.09
IVS d(mm)	0.77 ± 0.02	0.92 ± 0.06^a^	0.73 ± 0.05	0.83 ± 0.14
IVS s(mm)	1.16 ± 0.03	1.22 ± 0.10	1.19 ± 0.01	1.13 ± 0.14

a *p* < 0.05 vs Saline group.

b *p* < 0.05 vs CDDP group.

### Tumor Necrosis Factor-α Signal Pathway Is Involved in Cisplatin-Induced Cardiac Injury

**FIGURE 4:**
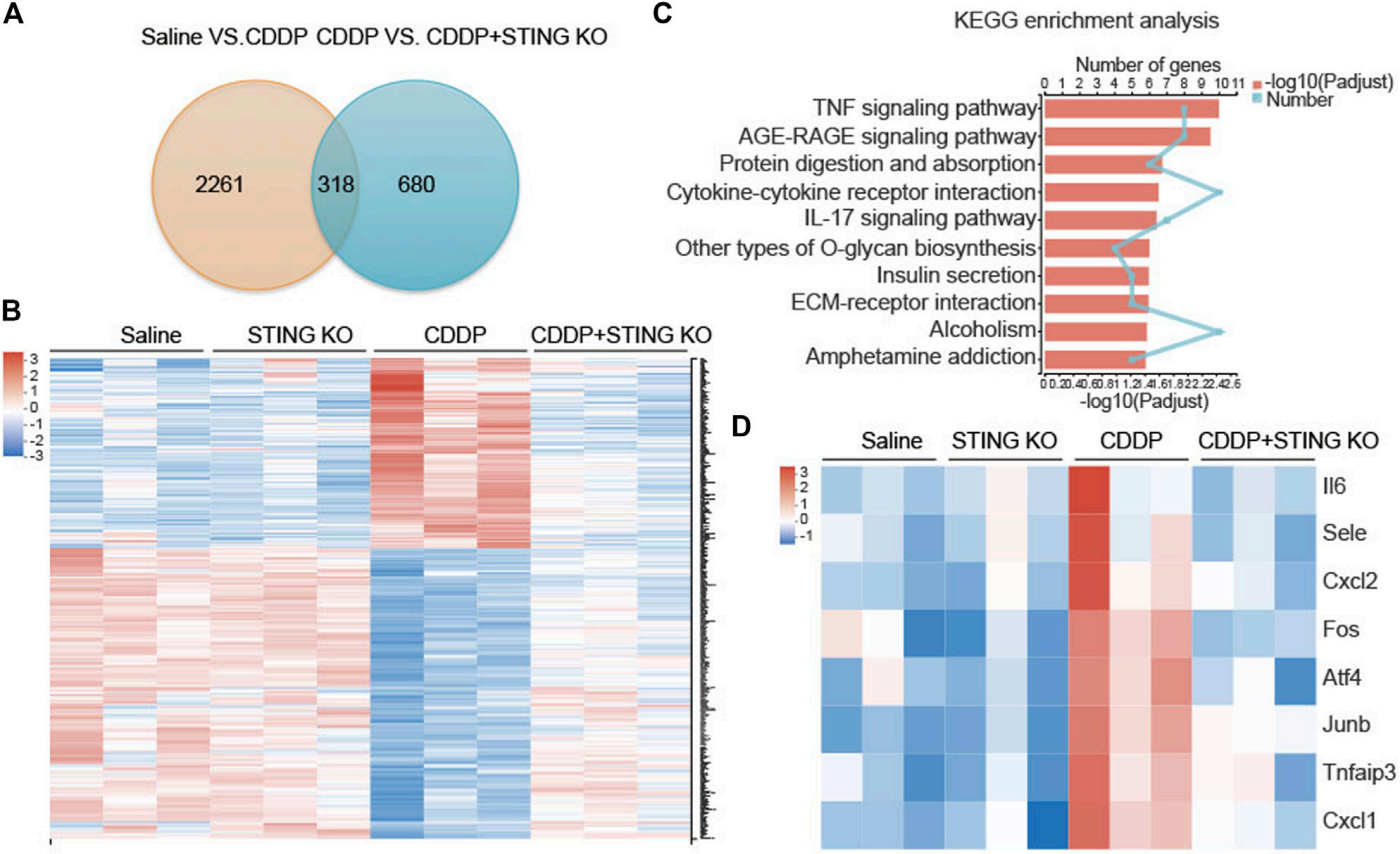
TNF signaling pathway was the most significantly enriched pathway identified by RNA-seq **(A)** Venn diagram of identified differentially expressed genes in the genome Saline vs. CDDP and the genome CDDP vs. STING KO + CDDP. **(B)** Differential gene-expression heat maps of identified genes were present in the genome Saline vs. CDDP and the genome CDDP vs. STING KO + CDDP **(C)** KEGG pathway enrichment analysis of the identified differentially expressed genes present in the genome Saline vs. CDDP and the genome CDDP vs. STING KO + CDDP. The 10 most significantly enriched pathways were shown **(D)** Differential gene-expression heat maps of identified genes were present in the TNF signaling pathway.

We performed RNA sequencing (RNA-seq) on cardiac tissues from wild-type (WT) and STING^−/-^ mice to better characterize the cardioprotective mechanism by deficiency of STING in CDDP-induced mice. Venn analysis showed 318 genes were present in the genome Saline vs. CDDP and the genome CDDP vs. STING KO + CDDP (Figure 4A). Heat map analysis with KEGG enrichment analysis confirmed a strong downregulation of TNF signaling pathway-related genes in cardiac tissues of STING^−/-^ mice (Figures 4B–D).

**FIGURE 5:**
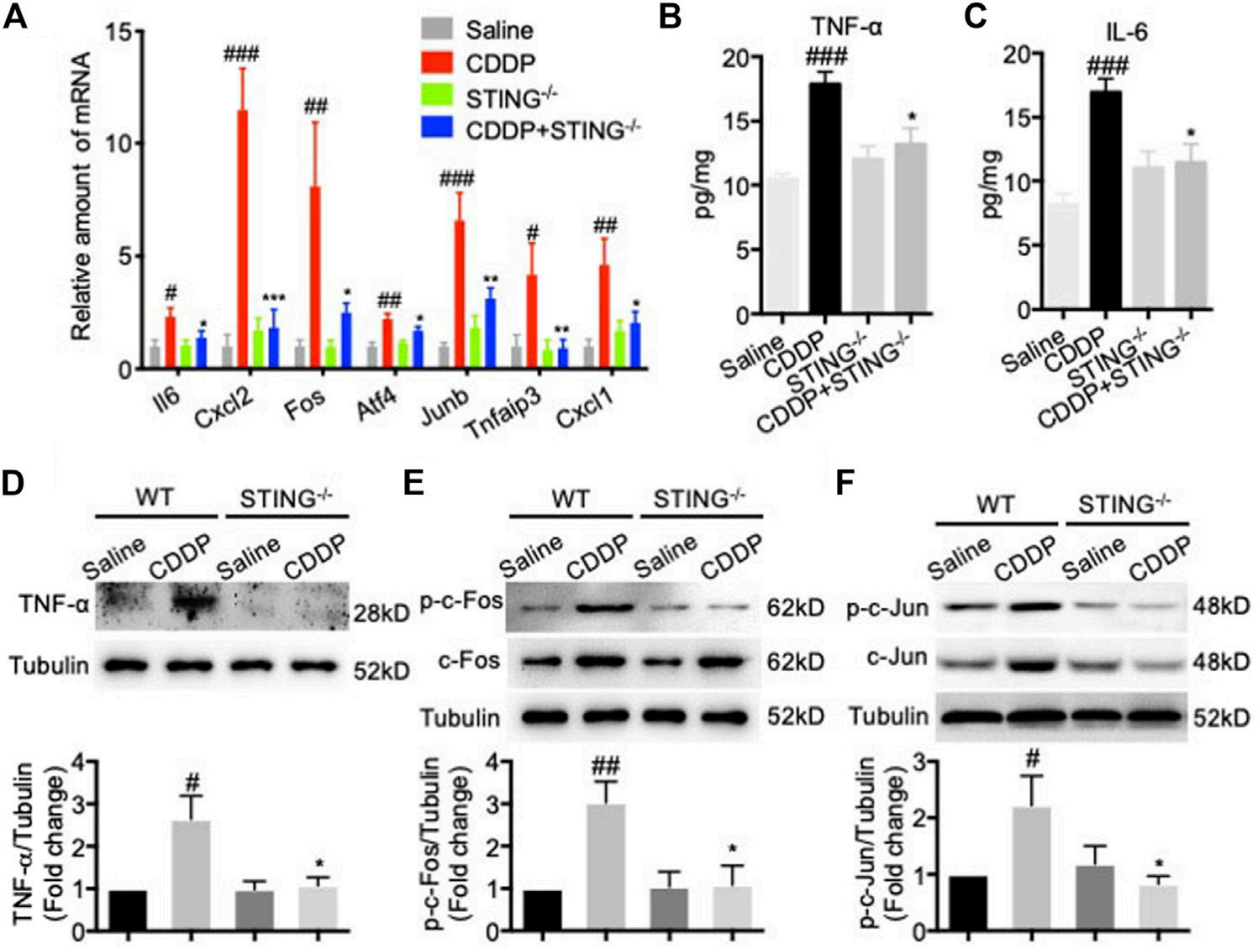
TNF signal pathway was involved in cisplatin-induced cardiac injury. **(A)** RT-qPCR was used to detect the expression of TNF signal pathway-related genes including Il6, Cxcl2, Fos, Atf4, Junb, Tnfaip3, and Cxcl1 in cardiac tissues **(B–C)** The expression of TNF-ɑ and IL-6 in cardiac tissues were detected by ELISA. **(D–F)** The protein expression of TNF-ɑ (D), p-c-Fos (E), c-Fos (E), p-c-Jun (F), c-Jun (F) in cardiac tissues were detected by Western blot (*n* = 6 in each group; ^#^ *p* < 0.05, ^##^ *p* < 0.01, ^###^ *p* < 0.001 vs Saline group; ^*^ *p* < 0.05, **p < 0.01, ***p < 0.001 vs. CDDP group).

The mRNA levels of Il6, Cxcl2, Fos, Atf4, Junb, Tnfaip3, and Cxcl1 and the protein levels of TNF-α and IL-6 in cardiac tissues were determined to identify the potential drivers of the hyperactive TNF signal pathway. Consistent with the results of RNA-seq, CDDP increased the mRNA levels of a variety of TNF signaling pathway-related genes, including Il6, Cxcl2, Fos, Atf4, Junb, Tnfaip3, and Cxcl1 in cardiac tissues, but these changes were dramatically reversed by *Sting* knockout (Figure 5A). Congruously, the increased protein levels of TNF-α and IL-6 induced by CDDP in cardiac tissues were reduced by *Sting* knockout (Figures 5B–D). The downstream of TNF signal pathway, AP-1, a heterodimer composed of c-Fos and c-Jun, was activated in cardiac tissues of CDDP-challenged mice but inactivated in cardiac tissues of CDDP-challenged STING^−/-^ mice (Figures 5E,F). These results indicated that the TNF signal pathway was involved in cisplatin-induced cardiac injury.

### Stimulator of Interferon Genes-Tumor Necrosis Factor-α-AP-1 Mediated Cisplatin-Induced Cardiomyocyte Injury

**FIGURE 6:**
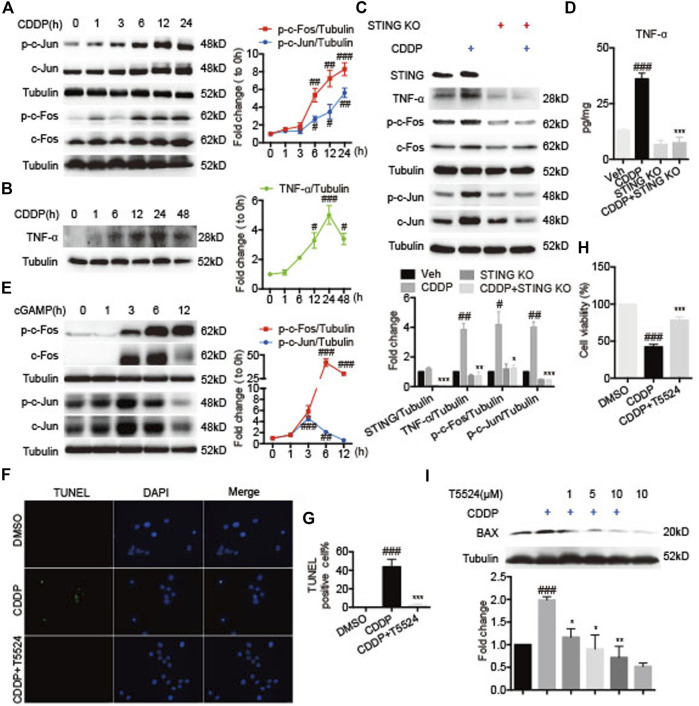
STING-TNF-α-AP-1 mediated cisplatin-induced cardiomyocyte injury. **(A–B)** HL-1 cells were incubated with CDDP (40 μM) for a time course, and then the total protein was collected. The protein levels of p-c-Fos, c-Fos, p-c-Jun, c-Jun, and TNF-ɑ were detected by Western blot. **(C)** HL-1 cells were incubated with CDDP (40 μM) for 24 h, and then the total protein was collected. The protein levels of STING, TNF-ɑ, p-c-Fos, c-Fos, p-c-Jun, c-Jun were detected by Western blot. **(D)** HL-1 cells were incubated with CDDP (40 μM) for 24 h, and the medium supernatant and the total protein were collected. The contents of TNF-ɑ and IL-6 in the medium supernatant were detected by ELISA and calibrated with protein concentration. **(E)** HL-1 cells were incubated with cGAMP (1 μg/ml) for a time course, and then the total protein was collected. The protein levels of p-c-Fos, c-Fos, p-c-Jun, c-Jun were detected by Western blot. **(F–G)** Representative images and quantification of TUNEL staining in HL-1 cells. HL-1 cells were pretreated with T5224 (10 μM) for 30 min and incubated with CDDP (40 μM) for 24 h. Cell apoptosis induced by CDDP was detected by TUNEL staining (Green: TUNEL positive cell, DAPI: nucleus; Scale Bar: 25 μm, ×400 magnification). **(H)** HL-1 cells were pretreated with T5224 (10 μM) for 30 min and incubated with CDDP (40 μM) for 24 h, and the cell viability of HL-1 cells was examined by CCK8 analysis. **(I)** HL-1 cells were pretreated with T5224 (1, 5, 10 μM) for 30 min and incubated with CDDP (40 μM) for 24 h, and the total protein was collected. The protein levels of BAX were detected by Western blot (*n* = 3 independent experiments; ^#^ *p* < 0.05, ^##^ *p* < 0.01, ^###^ *p* < 0.001 vs. Vehicle or DMSO group; ^*^ *p* < 0.05, **p < 0.01, ***p < 0.001, vs. CDDP group).

Next, we investigated the role of the STING-TNF-α-AP-1 axis in CDDP-induced cardiomyocytes injury. Exposed to CDDP, c-Jun and c-Fos were time-dependently phosphorylated in HL-1 cells (Figure 6A). Analogously, the protein level of TNF-α was time-dependently increased in HL-1 cells (Figure 6B). To further validate the regulatory role of STING in CDDP-induced activation of AP-1, we examined whether deficiency of STING inhibited the phosphorylation of AP-1 in CDDP-induced HL-1 cells. Expectedly, the protein and secretion level of TNF-α was suppressed (Figures 6C,D), and the phosphorylation of c-Jun and c-Fos was blocked by *Sting* knockout (Figure 6C). Of note, activation of STING by cGAMP promoted the phosphorylation and expression of c-Jun and c-Fos (Figure 6E). In addition, cardiomyocyte apoptosis induced by CDDP was attenuated by AP-1 inhibitor T5224 in HL-1 cells (Figures 6F,G). Moreover, the decrease in cell viability induced by CDDP was improved (Figure 6H), and the increased expression of BAX induced by CDDP was reversed by T5224 in HL-1 cells (Figure 6I). These results suggested that STING-TNF-α-AP-1 mediated CDDP-induced cardiomyocyte apoptosis.

## Discussion

This study demonstrated that STING played a crucial regulatory role in cisplatin-induced cardiotoxicity and demonstrated that cisplatin activated STING, which promoted the activation of the TNF-α-mediated AP-1 signaling pathway. However, deficiency of STING attenuated cisplatin-induced cardiac dysfunction and apoptosis *in vivo* and abolished cisplatin-induced cardiomyocyte apoptosis *in vitro*.

Apoptosis plays a key role in the development of chemotherapy-associated cardiopathy. Recently, accumulated studies have suggested that the mtDNA-cGAS -STING pathway proved critical to the progression of cardiovascular diseases (Mao et al., 2017; Cao et al., 2018; Sliter et al., 2018; Li et al., 2019; Hu et al., 2020; Huang et al., 2020; Zhang et al., 2020). In the present study, our results showed that STING was an essential regulator for cisplatin-induced cardiotoxicity. In our *in vivo* experiments, STING and TBK1 in cardiac tissues were phosphorylated by intraperitoneal injection with cisplatin. The deficiency of STING reduced the phosphorylation of TBK1 in cardiac tissue.

Moreover, deficiency of STING attenuated not only cisplatin-induced cardiac dysfunction and the increase in serum CK-MB but also improved cisplatin-induced myocardial fiber disorder and cardiomyocyte apoptosis. In *vitro* experiments, deficiency of STING abolished cisplatin-induced apoptosis of HL-1 cells. These results indicated that suppression of STING protected against cardiotoxicity induced by cisplatin.

Recent studies suggested that targeting the TNF signal pathway might comprise a viable therapeutic strategy to reduce disease burden in ischaemic vascular disease progression, particularly endothelial dysfunction, chronic inflammation, and atherosclerotic plaque development (Hu et al., 2015; Kojima et al., 2016; Nash et al., 2019). Heat map analysis with KEGG enrichment analysis in RNA-seq from cardiac tissues confirmed a strong correlation between cisplatin-induced cardiotoxicity and TNF signaling pathway, and deficiency of STING alleviated cisplatin-induced cardiac dysfunction, which is significantly correlated with the inactivation of the TNF signal pathway. Consistently, the expression of TNF-α and the phosphorylation of c-Jun and c-Fos were increased, but these were reversed by deficiency of STING. cGAMP bind to the endoplasmic reticulum (ER)-localized STING, which promotes STING dimerization and translocation from ER to the endoplasmic reticulum-Golgi intermediate compartment (ERGIC) (Sliter et al., 2018; Ablasser and Chen, 2019; Gui et al., 2019; Shang et al., 2019; Zhang et al., 2019). Subsequently, STING recruits and activates TBK1 to initiate an IRF3-dependent innate immune response, resulting in the production of IFNs and a NF-κB-dependent inflammatory response, which leads to the production of pro-inflammatory factors, including TNF-α, IL6, IL1β, etc. (Burdette et al., 2011; Bai and Liu, 2019; Zhang et al., 2019; Hu and Shu, 2020). Our results showed that the activation of AP-1 was dependent on an increased expression of TNF-α. Intriguingly, the expression of c-Jun and c-Fos was increased when exposed to both cisplatin and cGAMP, indicating that AP-1 might be mediated by the STING-NF-κB signaling pathway.

## Conclusion

Our study found that the STING-TNF-α-AP-1 axis contributed to cisplatin-induced cardiotoxicity by triggering cardiomyocyte apoptosis and deficiency of STING attenuated cisplatin-induced cardiac dysfunction. Moreover, STING may be a potential therapeutic target for preventing the progression of chemotherapy-associated cardiovascular complications.

## Data Availability

The data presented in the study are deposited in the GEO repository, accession number:GSE181749.
